# Prevalence and predictive parameters of external root resorption caused by retained wisdom teeth

**DOI:** 10.1007/s00784-024-05964-0

**Published:** 2024-10-09

**Authors:** Frederik Baensch, Wilhelm Meißner, Lena Will, Martin Kunkel

**Affiliations:** 1 Department of Oral and Maxillofacial Surgery, University of Bochum Medical School, Universitätsklinikum Knappschaftskrankenhaus Bochum, In der Schornau 23-25, D-44892 Bochum, Germany; 2 Institute of Diagnostic and Interventional Radiology, Neuroradiology and Nuclear Medicine, Universitätsklinikum Knappschaftskrankenhaus Bochum, In der Schornau 23-25, D-44892 Bochum, Germany

**Keywords:** Retained teeth, Impacted teeth, Root resorption, Tooth eruption, Wisdom teeth, Third molars

## Abstract

**Objectives:**

The aim of this study was to estimate the prevalence and severity of external root resorption (RR) caused by retained third molars (M3), to compare the sensitivity of panoramic radiography (PAN) and cone beam computed tomography (CBCT) and to determine predictive factors for root resorption.

**Materials and methods:**

In a retrospective cross-sectional analysis, we included patients (*N* = 367) who underwent PAN and CBCT imaging between December 2017 and July 2019. Previous orthodontic treatment, age, gender, superimposition of second molars (M2) and M3 on PAN, retention depth, inclination angle and vertical level of contact with the M2 were used as predictor variables. The outcome variable was RR of the M2, graded according to Ericson et al. [1]. Subgroup analyses compared patients with and without suspected resorption in the PAN.

**Results:**

While less than 5% of PANs suggested RR associated with M3, CBCT showed RR in 20% of all M2 with adjacent retained M3. The angle of inclination of M3, patient age and vertical level of molar contact emerged as predictive parameters, with mesial inclination, older age and deeper retention associated with increased severity of M2.

**Conclusion:**

Within the limitations of our study, these data confirm the poor performance of PAN in the diagnosis of RR. CBCT may be helpful in detecting RR in mesioangulated and deeply retained M3 in elderly patients, even when PAN did not suggest pathology.

**Clinical relevance:**

Our study may help to decide whether CBCT should be considered prior to M3 surgery.

## Introduction

Physiologically, teeth erupt along their longitudinal axis until they reach the occlusal plane, guided by anatomical structures such as the tongue, cheeks and lips [1]. During the eruption of permanent incisors, canines and premolars, the roots of each primary tooth are resorbed. Therefore, external root resorption (RR) can be considered as a physiological process. However, lack of space, atopic position or inclination of the retained tooth can lead to pathological RR of adjacent permanent teeth.

**Fig. 1:**
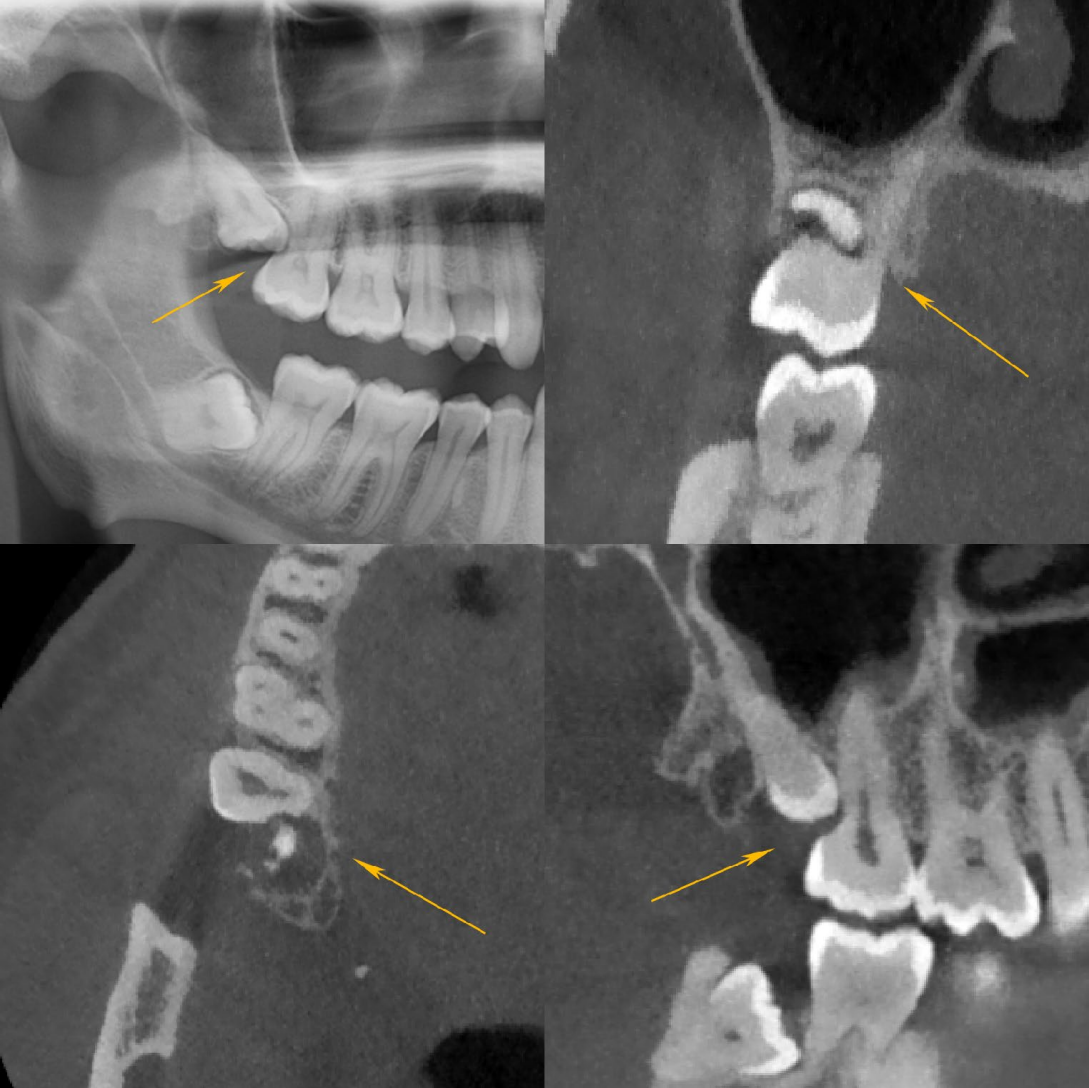
Mild resorption of the palatal root of the upper right M2. Superimposition and mesioangulation (31 degrees). RR affecting only the outer half of the dentine. Panoramic radiograph (**a**, top left), CBCT frontal slice (**b**, top right), axial slice (**c**, bottom left) and sagittal slice (**d**, bottom right). The arrows are pointing to the resorption zone

**Fig. 2:**
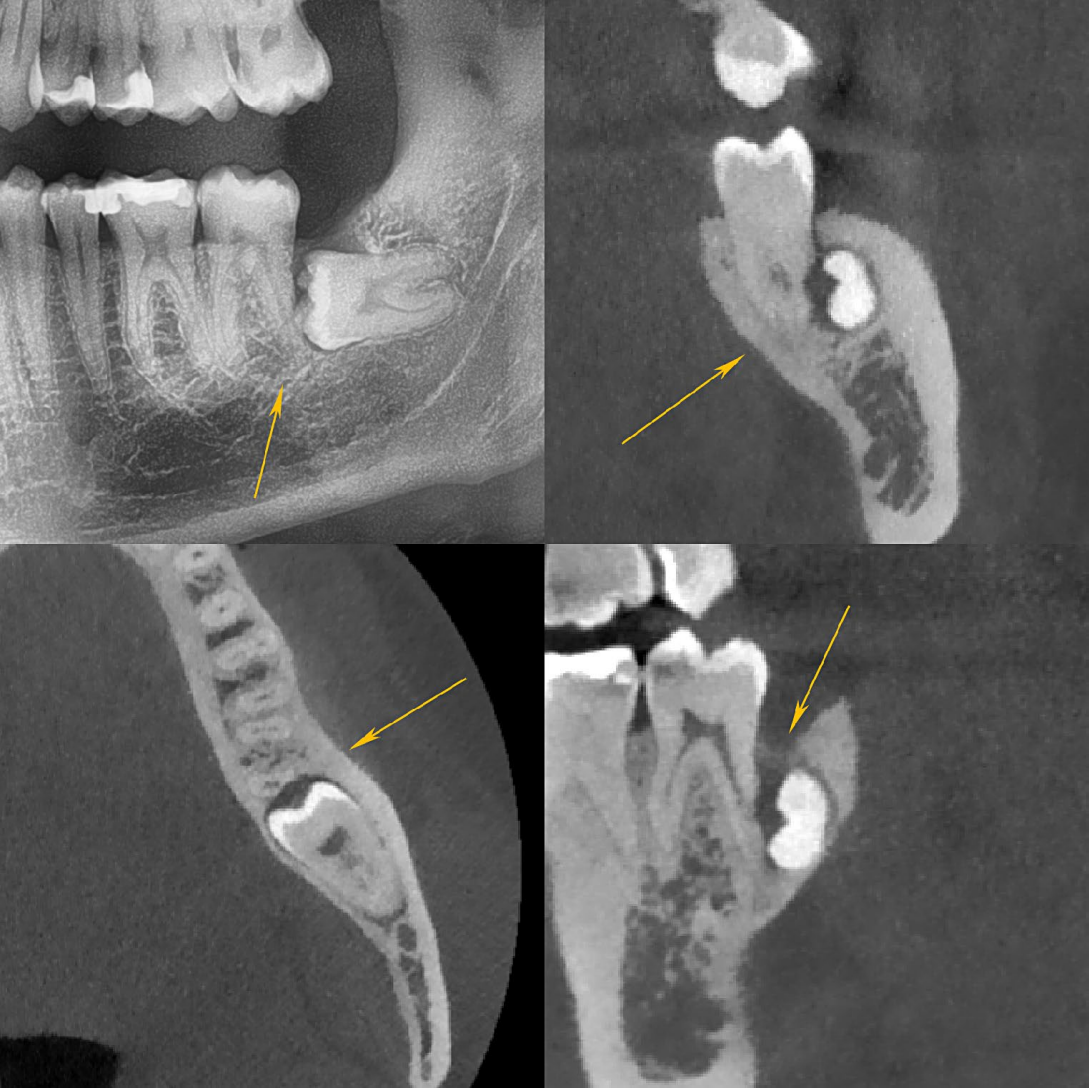
Moderate resorption of the distal root of the lower left M2. Superimposition and horizontal inclination. RR reaches the inner half of the dentine layer. Panoramic radiograph (**a**, top left), CBCT frontal slice (**b**, top right), axial slice (**c**, bottom left) and sagittal slice (**d**, bottom right). The arrows are pointing to the resorption zone

**Fig. 3:**
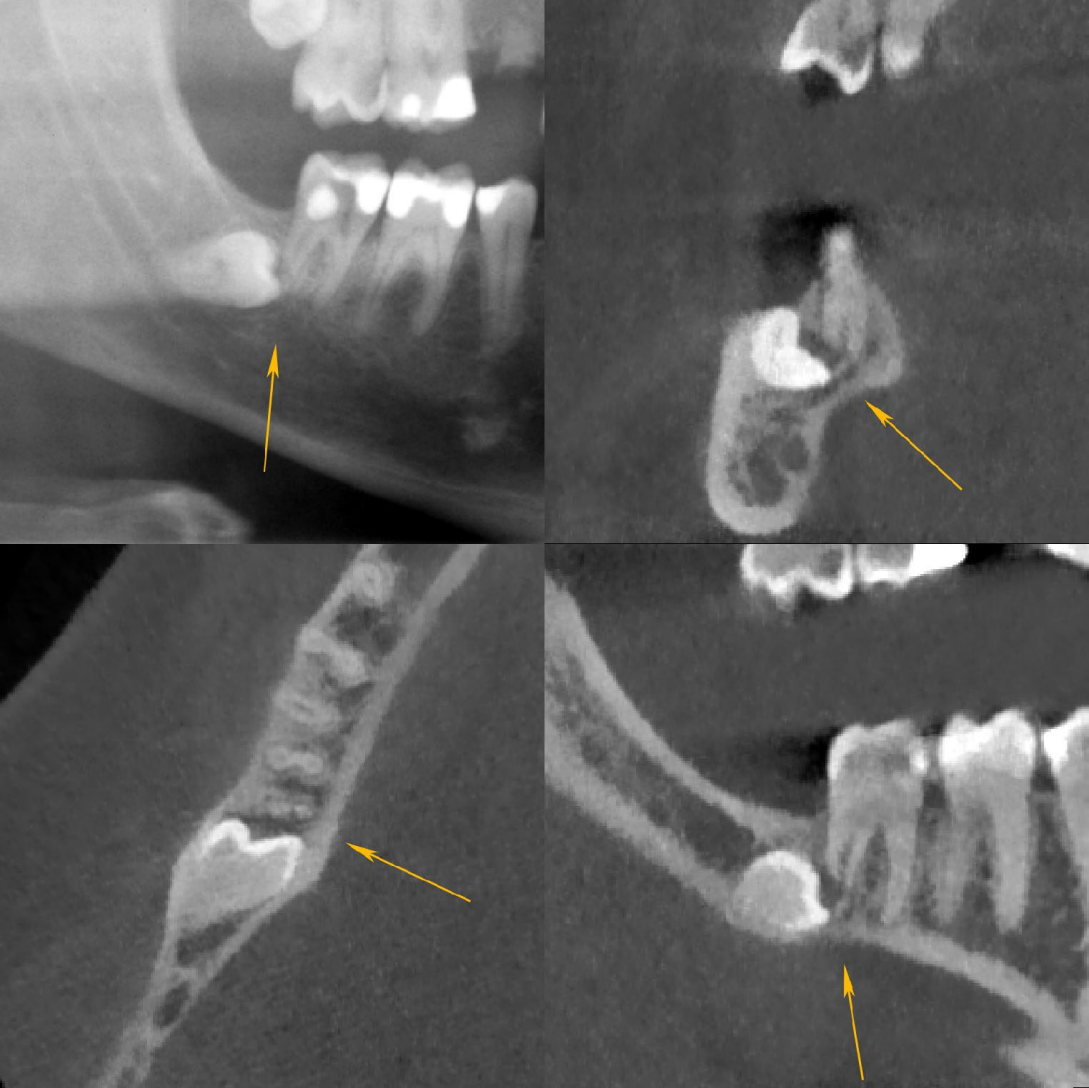
Severe resorption of the distal root of the lower right M2. Superimposition and mesioangulation (63 degrees). Dentin resorption leads to exposure of the pulp. Panoramic radiograph (**a**, top left), CBCT frontal slice (**b**, top right), axial slice (**c**, bottom left) and sagittal slice (**d**, bottom right). The arrows are pointing to the resorption zone

The severity of RR is commonly graded according to Ericson et al. [2]. This classification distinguishes between mild (the outer half of dentin is resorbed, see Fig. 1), moderate (the inner half of dentin is resorbed, see Fig. 2) and severe (the root canal is exposed, see Fig. 3) stages of resorption.

Although the topic of RR in general has been addressed by some authors for several decades [3–7], RR caused by retained teeth has recently become a focus of interest due to the progress in radiological imaging techniques, in particular the dissemination of high-quality three-dimensional dental scans. Cone beam computerized tomography-based (CBCT) studies have shown that approximately 30% of patients with retained maxillary canines have RR of their lateral (or central) incisors [8–13], while other studies have reported rates of up to 70% [14, 15].

While the prevalence of RR caused by retained canines has been frequently addressed and plausible data has been published in recent years, there is insufficient knowledge about the prevalence of RR of second molars (M2) caused by retained third molars (M3). Some studies suggest a prevalence of up to 50% in mesially inclined M3 [16]. However, it seems highly unlikely that these extraordinarily high RR rates can be extrapolated to the general population or even to a population at risk due to mesial inclination.

Although mesial angulation and horizontal inclination of M3 have been suggested to increase the risk of RR [17], their value as potential predictive parameters for M2 RR has not been systematically investigated. Therefore, we also investigated whether M3 angulation, retention depth, tooth contact position, age or previous orthodontic treatment were associated with M2 RR and compared CBCT findings in patients with and without suggested M3 RR based on their panoramic radiographs (PAN).

Some hypotheses suggest that mechanical contact and/or pressure are relevant for the development of RR [18, 19]. In this context, the extent of RR and, therefore, the clinical consequences for the patient could be related to the angle between the retained tooth and the adjacent tooth (inclination), the localization of the contact zone (distance from the contact point and the enamel-cement junction) and the depth of retention. However, in many cases of pathological/critical inclination and retention, there is no RR at all. Furthermore, the relevance of both parameters, depth of retention and point of contact, has been questioned by others [20].

## Materials and methods

### Study design/sample

All patients attending our clinic between 1 December 2017 and 30 November 2019 were screened for eligibility for this retrospective cross-sectional study.

The study was performed in line with the principles of the Declaration of Helsinki. It was approved by the institutional review board of Ruhr University, Bochum, Germany (Reg.-Nr. 18-6652-BR) in view of the retrospective nature of the study and all the procedures being performed were part of the routine care.

Patients were included if both a CBCT scan (NewTom 5G XL, Quantitative Radiology, Verona, Italy; field of view (FoV) of 12 × 8 cm and a voxel size of 0.2 mm) and a PAN (Sirona Orthophos SL 2D, Bensheim, Germany) within a short time interval (up to 6 months before or after the CBCT) were available. Cases were excluded if there was no retained M3 or if the quality of either the CBCT or the PAN was insufficient. Image quality was assessed by both the radiologist’s report of the scan and the study investigator. A sharp contrast between the root canal and the dentin of the retained and adjacent tooth was used as a parameter of image quality. Both CBCT and PAN scans were examined by a single observer, who was trained in maxillofacial surgery and had four years of clinical experience in assessing dental radiographic images. The findings were corroborated by the attending radiologist’s report of the scan.

To assess the diagnostic value of PAN, patients were divided into two subgroups: Group 1 showed signs of potential RR in the PAN (overlapping of the M3 crown and the M2 root(s) and/or visible resorption of the M2), while group 2 had no signs of RR in the PAN.

### Study variables

Patients’ age, gender and previous orthodontic treatment were used as systemic predictor variables, while the superimposition of M2 and M3 in the PAN, retention depth, inclination angle and the vertical level of contact with the M2 were used as local predictor variables.

The outcome variable was RR of the M2, graded according to Ericson et al. [2].

**Fig. 4:**
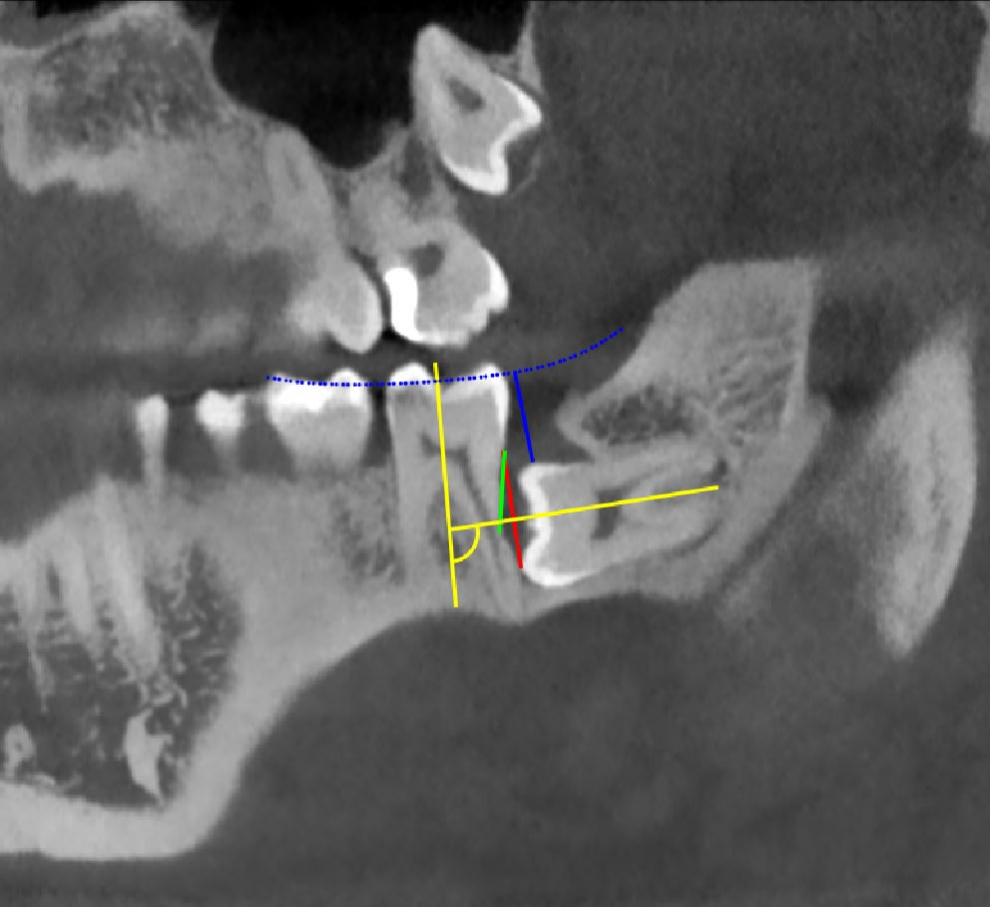
Example of CBCT analysis: Blue line (dotted): Occlusal plane Blue line: Retention depth (shortest distance from retained M3 to the occlusal plane), 7 mm Red line: Contact point (closest point of contact between M2 and M3 measured from the enamel-cement junction of M2), 9 mm Green line: Point of maximum resorption (measured from the enamel-cement junction of M2 to the area of greatest dentin loss based on apex cross-section, 6.5 mm Yellow lines: Angle between the tooth axes of M2 and M3, 94 degrees

The angle of inclination was determined as the angle between the longitudinal axes of M2 and M3. The point of contact was determined by measuring the shortest distance between the enamel-cement junction of M2 and the point of contact between the cusp of M3 and the root of M2. The depth of retention was determined as the shortest (perpendicular) distance between the occlusal plane and M3. The point of maximum resorption (POMR) was determined as the shortest distance between the enamel-cement junction of M2 and the area of greatest dentin loss. A representative image of CBCT analysis can be found in Fig. 4.

The degree of RR was categorized according to Ericson et al. [2]: An intact surface was reported as no RR. Mild RR describes loss of less than half of the dentin layer. Once the RR reached the inner half of the dentin, it was considered as moderate RR. An exposed pulp was categorized as severe RR.

### Statistical analysis

IBM SPSS Statistics for Mac, version 22, was used for all calculations. Descriptive data (absolute and relative frequencies, means, ranges and standard deviations) were compiled. Correlation analysis was performed using Spearman´s analysis [21, 22]. Nominal differences between groups as defined by the predictor variables were calculated using the chi-squared test of independence [23], first established by Pearson [24], while ordinal and metric differences were determined using the Mann and Whitney two-tailed non-parametric U-test [25]. Due to the exploratory nature of this study, alpha adjustment was not performed and p-values should be considered descriptive [26].

### Sample size calculation

A priori sample size calculation for two-sided Mann-Whitney-U-Test indicated that a minimum of 344 M3 was required to achieve a power of 95%, an alpha of 5% and an effect size of 0.5 to compare M3 from group 1 and 2. This was based on the sensitivity of the PAN analysis as stated by Oenning et al. [27]. A sample size of 319 M3 was required for two-sided correlation testing, assuming a correlation coefficient of ρ = 0.2, a power of 95% and an alpha of 5%.

### Independency of observations

Since RR on one side is not independent of resorption on another side (Chi-Square test: *p* < 0.001), we used both the patient and the individual tooth as the statistical observation unit for the evaluation. Both approaches have some disadvantages (see discussion section).

For the analysis per patient, only one M3 was selected per patient (*n* = 367). In order to achieve true randomisation and therefore independency of observation, a random numeric value was assigned to each of the 914 M3. The dataset was then ordered by the random value, and duplicate cases were eliminated by using the patient ID. This process ensured that only one M3 per patient was randomly selected without favoring either a higher level of RR or a specific jaw.

### Homogeneity of variance

In order to account for the violation of the assumption of homogeneity of variances in some of our study variables, we elected to employ the non-parametric Mann-Whitney U-Test [28].

## Results

### Patients and diagnoses

From 1 December 2017 to 30 November 2019, a total of 2253 patients received a PAN in our clinic. Of these patients, 367 also received a CBCT scan, which identified at least one retained M3 in proximity to other teeth. Among these patients, 914 retained M3s were identified (433 in the maxilla and 481 in the mandible).

The study population comprised 174 male patients (47.4%) and 193 female patients (52.6%). The mean age of all patients was 26.97 ± 16.31 years, with a range of 11 to 87 years. Of the patients, 214 (58.3%) had previously undergone orthodontic treatment. A total of 13 patients (3.5%) exhibited dental abnormalities, including hyperdontia (11 patients, 2.9%) and hypodontia (3 patients, 0.8%).

In 160 patients (43.6%), CBCT was conducted primarily to rule out RR, while 112 patients (30.5%) underwent three-dimensional diagnostics due to proximity to the inferior alveolar nerve (IAN). In the remaining 95 patients, the indication for CBCT imaging was not related to M3 aspects (25.9%). Of the 461 M3s (50.4%) that exhibited superimposition of M2 and adjacent M3 in the PAN, 49.4% were maxillary M3s and 51.4% mandibular M3s. The remaining 453 M3s (49.6%) were assigned to group 2.

### Study variables

- Distribution of predictor variables in the study subgroups.

There was no notable difference (*p* = 0.627) in mean age between group 1 (26.5 ± 15.7 years) and group 2 (27.5 ± 16.9 years). Furthermore, there was no observable difference in gender distribution (*p* = 0.093) or previous orthodontic treatment (*p* = 0.643). In addition, the number of maxillary and mandibular teeth was comparable between the two groups (group 1: 214 maxillary and 247 mandibular teeth; group 2: 219 maxillary and 234 mandibular teeth). There was no relevant difference in the depth of retention (*p* = 0.646) between the two groups. Nevertheless, a marginal difference was observed when the maxilla was considered separately. (7.55 ± 2.53 mm in group 1 vs. 7.13 ± 2.8 mm in group 2; *p* = 0.038).

While there was no decisive difference in the angle of inclination in the maxilla (*p* = 0.245), there was a significantly greater angle of inclination for mandibular M3 with superimposition in the PAN (group 1: 45.29 ± 28.71 degrees vs. group 2: 30.52 ± 33.25 degrees; *p* < 0.001). The proportion of mesioangulated M3 (angle between 10 and 80 degrees) was 60.3% in group 1 compared to 49.0% in group 2 (*p* = 0.001).

The contact point of superimposed M3 observed to be situated at a greater distance from the enamel-cement junction M2 (4.72 ± 3.1 mm in group 1 vs. 4.1 ± 3.05 mm in group 2; *p* = 0.005). For a detailed breakdown of the parameters, please refer to Table 1.

**Table 1:** Predictor variables in the study: overall and according to maxillary and mandibular M3

	Group 1		Group 2		
	N	Mean; SD; range	N	Mean; SD; range	p
All M3					
Age [years]	461	26.5 ± 15.7 [11; 87]	453	27.5 ± 16.9 [11; 85]	0.627
Depth of retention [mm]	461	5.8 ± 3 [-0.5; 17]	453	5.8 ± 3 [0; 31.5]	0.646
Angle of inclination	461	25.4 ± 34.7 [-79.5; 113]	453	16.8 ± 31.4 [-51.5; 172.5]	<0.001
Point of contact in [mm]	421	4.7 ± 3.1 [0; 15.5]	343	4.1 ± 3.1 [0; 13.5]	0.005
Maxillary M3					
Age [years]	214	27.3 ± 16.0 [12; 83]	219	23.3 ± 14.2 [11; 85]	<0.001
Depth of retention in [mm]	214	7.6 ± 2.5 [1.5; 17]	219	7.1 ± 2.8 [1.5; 31.5]	0.038
Angle of inclination	214	2.5 ± 25.8 [-79.5; 64.5]	219	2.1 ± 21.2 [-51.5; 126]	0.245
Point of contact in [mm]	207	5.9 ± 3 [0; 15.5]	201	5 ± 2.8 [0; 13.5]	0.001
Mandibular M3					
Age [years]	247	25.7 ± 15.5 [11; 87]	234	31.4 ± 18.2 [12; 84]	<0.001
Depth of retention in [mm]	247	4.3 ± 2.5 [-0.5; 12.5]	234	4.5 ± 2.6 [0; 18]	0.497
Angle of inclination	247	45.3 ± 28.7 [-48; 113]	234	30.5 ± 33.3 [-47; 172.5]	<0.001
Point of contact in [mm]	214	3.6 ± 2.7 [0; 11]	142	2.9 ± 3 [0; 11]	0.002

- Per molar-analysis: Outcome parameters in the study groups.

The occurrence of RR was found to be 50% more prevalent in cases where superimposition was present in the PAN (Group 1: 24% vs. Group 2: 16%; *p* = 0.003). This finding is consistent when all M3 are considered, as well as when maxillary and mandibular M3 are analysed separately. The rates of RR were 28% for maxillary M3 and 21% for mandibular M3, with p-values of 0.04 and 0.024, respectively. Mesioangulation also led to higher RR rates. The lowest RR rate was observed in cases with neither superimposition nor mesioangulation (13.9%), while mesioangulated M3 without superimposition exhibited similar overall RR rates to non-mesioangulated M3 with superimposition (18.9% vs. 19.7%). Superimposition appeared to have a greater effect on the severity of RR. The non-mesioangulated M3 with superimposition exhibited a 5.8% incidence of moderate/severe RR, in comparison to a 1% rate for the mesioangulated M3 without superimposition. The highest rate of RR was observed in the M3 with both mesioangulation and superimposition, with a 27.3% incidence, including 7.2% of moderate/severe RR.

In general, the degree of RR was found to be more pronounced when superimposition was present in the PAN. The proportion of moderate to severe RR was 28% in group 1 and only 12% in group 2 (*p* = 0.001) for all M3, 35% vs. 14% (*p* = 0.021) for maxillary M3 and 19% vs. 10% (*p* = 0.020) for mandibular M3.

PANs were able to detect RR in only 7 teeth (0.8%) and could only depict RR in the mandible. Of these, 4 were later classified as mild RR on CBCT, and 3 were classified as severe. Thus, only 3.8% of all RR (7/186) and only 7.5% (3/40) of moderate or severe RR were detected by PAN imaging. Please refer to Table 2 for a detailed breakdown of the parameters.

**Table 2:** Outcome variables in the study: overall and according to maxillary and mandibular M3

	Group 1		Group 2		
	N	Mean; SD; range	N	Mean; SD; range	p
All M3					
RR	461	24.3%	453	16.3%	0.003
Level of RR	461		453		
•No resorption	349	75.7%	379	83.7%	
•Slight	81	17.6%	65	14.3%	
•Moderate	17	3.7%	5	1.1%	
•Severe	14	3.0%	4	0.9%	
Point of maximum resorption in [mm]	110	5.2 ± 2.9 [0.5; 11.5]	72	4.8 ± 2.5 [0.5; 10.5]	0.450
Maxillary M3					
RR	214	28.0%	219	19.6%	0.040
Level of RR	214		219		
•No resorption	154	72.0%	176	80.4%	
•Slight	39	18.2%	37	16.9%	
•Moderate	15	7.0%	4	1.8%	
•Severe	6	2.8%	2	0.9%	
Point of maximum resorption in [mm]	60	6.7 ± 2.4 [1.5; 11.5]	43	5.4 ± 2.3 [0.5; 9.5]	0.012
Mandibular M3					
RR	247	21.1%	234	13.2%	0.024
Level of RR	247		234		
•No resorption	195	78.9%	203	86.8%	
•Slight	42	17.0%	28	12.0%	
•Moderate	2	0.8%	1	0.4%	
•Severe	8	3.2%	2	0.9%	
Point of maximum resorption in [mm]	50	3.4 ± 2.5 [0.5; 10]	29	3.9 ± 2.6 [0.5; 10.5]	0.397

- Per patient-analysis: Outcome parameters in the study groups.

The outcome parameters in each study group were additionally analysed on a per patient level.

This involved examining 92 upper-right M3s, 79 upper-left M3s (171 maxillary M3s), 101 lower-left M3s and 95 lower-right M3s (196 mandibular M3s).

RR is decisively more frequent in group 1 (*p* = 0.003) and more severe (*p* = 0.002) with a significantly higher angle of inclination (*p* < 0.001) when all eligible M3 are examined.

With regard to the maxillary M3s only, RR trended to be more frequent in group 1 (*p* = 0.07), with the level of RR being more severe (*p* = 0.045). Furthermore, the point of contact was located more apically (*p* = 0.003).

When examining only mandibular M3, the prevalence and severity of RR were found to be significantly higher for group 1 (*p* = 0.019 and *p* = 0.016). Furthermore, the angle of inclination was found to be greater (*p* < 0.001). Patients older than 28 years were significantly more likely to show at least one case of RR compared to younger patients (31.1% vs. 20.5%, *p* = 0.022).

- Per molar-analysis: Correlation between predictor variables and RR.

In the per molar-analysis, a weak but significant correlation was found between inclination angle and RR in both study groups (group 1: *r* = 0.099; *p* = 0.033, group 2: *r* = 0.149; *p* = 0.002). This indicates that M3 with a more mesially inclined angle exhibited higher rates of RR. This is also true when maxillary and mandibular M3 are considered separately. Mesio-angulation is generally correlated with both higher rates of RR (*r* = 0.085, *p* = 0.010) and increased severity of RR (*r* = 0.089, *p* = 0.007), although the correlation coefficients are weak. In contrast, the contact point demonstrated a correlation with RR solely within group 2 (*r* = 0.225 and *p* = 0.007) in that a deeper contact point was associated with a higher frequency and severity of RR. There was no apparent correlation between gender and RR. However, advanced age appears to have a slight correlation with RR prevalence (*r* = 0.173 and *p* < 0.001) and RR severity (*r* = 0.189 and *p* < 0.001) when there is M2 superimposition in the PAN. However, when there was no superimposition in the PAN, the correlation was even lower (*r* = 0.094 and *p* = 0.046) and there was no association with RR severity.

Neither previous orthodontic treatment nor depth of retention demonstrated any predictive value for RR. When comparing the predictor variables of cases with moderate or severe RR with M3 with no or mild RR, there was a relevant difference for depth of retention, contact point and patient age (all *p* < 0.001).

A summary of parameters can be found in Table 3.

**Table 3:** Per molar-correlation analysis for predictor and outcome variables (Spearman rank-correlation)

All M3		Group 1 (*n* = 461)		Group 2 (*n* = 453)	
		RR	Level of RR	RR	Level of RR
Angle of inclination	r	0.099	0.092	0.149	0.146
	p	0.033	0.049	0.002	0.002
Age	r	0.173	0.189	0.087	0.094
	p	0.000	0.000	0.065	0.046
Maxillary M3		Group 1 (*n* = 214)		Group 2 (*n* = 219)	
		RR	Level of RR	RR	Level of RR
Angle of inclination	r	0.207	0.201	0.179	0.174
	p	0.002	0.003	0.008	0.010
Age	r	0.126	0.150	0.193	0.204
	p	0.066	0.028	0.004	0.002
Mandibular M3		Group 1 (*n* = 247)		Group 2 (*n* = 234)	
		RR	Level of RR	RR	Level of RR
Point of contact in mm	r	-0.084	-0.064	0.225	0.233
	p	0.220	0.349	0.007	0.005
	n	214	214	142	142
Angle of inclination	r	0.173	0.173	0.289	0.287
	p	0.006	0.006	<0.001	<0.001
Depth of retention in mm	r	-0.125	-0.113	-0.156	-0.148
	p	0.049	0.077	0.017	0.024
Age	r	0.204	0.215	0.033	0.037
	p	0.001	0.001	0.611	0.571

- Per patient analysis: Correlation between predictor variables and RR (Table 4).

**Table 4:** Per patient-correlation analysis for predictor and outcome variables (Spearman rank-correlation)

All M3		Group 1 (*n* = 188)		Group 2 (*n* = 179)	
		RR	Level of RR	RR	Level of RR
Age	r	0.198	0.217	0.075	0.086
	p	0.007	0.003	0.317	0.254
Maxillary M3		Group 1 (*n* = 91)		Group 2 (*n* = 80)	
		RR	Level of RR	RR	Level of RR
Angle of inclination	r	0.236	0.215	0.207	0.195
	p	0.024	0.040	0.065	0.083
Age	r	0.310	0.310	0.196	0.212
	p	0.003	0.003	0.081	0.059
Mandibular M3		Group 1 (*n* = 97)		Group 2 (*n* = 99)	
		RR	Level of RR	RR	Level of RR
Angle of inclination	r	0.151	0.147	0.211	0.207
	p	0.139	0.151	0.036	0.039
Depth of retention in mm	r	-0.221	-0.179	-0.173	-0.160
	p	0.030	0.080	0.088	0.113

In the per-patient analysis, a weak but significant correlation was found between patient age and RR as well as level of RR in group 1 when examining all M3s (RR: *r* = 0.198; *p* = 0.007; level of RR *r* = 0.217, *p* = 0.003). As for maxillary M3, patients in group 1 showed a significant correlation between the angle of inclination and RR as well as level of RR (RR: *r* = 0.0236, *p* = 0.024; level of RR: *r* = 0.215, *p* = 0.040). When examining mandibular M3, we found a significant correlation between the depth of retention and RR for patients in group 1 (*r* = -0.221, *p* = 0.030), with a shallow depth of retention increasing RR probability. As for group 2, we found a significant correlation between the angle of inclination and both RR and level of RR (RR: *r* = 0.211, *p* = 0.036; level of RR: *r* = 0.207, *p* = 0.039).

In line with the findings at the molar level, previous orthodontic treatment was not found to be predictive of RR.

- Homogeneity of variance.

The variance of the depth of retention and the point of contact is homogeneous among all observation scales, while the variance of point of maximum RR is homogeneous when examining maxillary and mandibular M3 separately. The variance of the angle of inclination is only homogeneous when observing mandibular M3.

## Discussion

It has been well documented for over two decades that root resorption (RR) caused by retained teeth is a common phenomenon. However, although the retention of M3 is strikingly more prevalent than retained canines (approx. 85% and 2.5% respectively), the issue of RR of neighboring teeth has been investigated way more thoroughly in the context of retained canines in the past years.

In 2000, Ericson and Kurol described RR of the lateral incisors in 38% of cases of ectopic maxillary canines and suggested direct contact between the incisors and the ectopic canine as well as the angle of inclination of the latter as risk factors [7]. Their study used fan beam computerized tomography, but still shows similar results to contemporary CBCT studies [13, 29].

M3-related studies have reported RR rates ranging from 20.2 to 52.5% [16, 17, 20, 27, 30–35]. The discrepancy in the reported RR rate can be largely attributed to differences in the inclusion criteria. Some studies included only horizontally or mesioangularly impacted mandibular M3s [16, 20], while others excluded M3s without physical contact to the adjacent M2 [17, 30]. It can be reasonably assumed that the particularly high RR rates observed in some studies are most likely explained by selection bias. Studies with less restrictive inclusion criteria have yielded similar results to those observed in our study, with mesioangulation and physical contact being the predominant risk factors.

Half of the studies utilised data from tertiary centres [17, 20, 31, 32, 34], while the remainder employed data from radiological archives [33, 35] or dental schools [16, 27]. Only one study employed data from a single private dental practice [30]. None of these studies compared high-risk M3 (horizontal or mesioangular inclination) to low-risk M3.

In contrast to the majority of the aforementioned studies, we included retained M3 with a variety of indications for radiographic imaging, provided that both a CBCT and PAN scan were available. Consequently, only 160 patients (43.6%) underwent three-dimensional imaging to exclude RR, while the remaining patients underwent imaging for other indications (mainly proximity to the IAN).

It was anticipated that this would reduce the selection bias that is unavoidable when only M3 imaging cases referred to a tertiary centre are included. In order to further reduce selection bias, patients who did not present with M3-related indications were also included, provided that both a PAN and a CBCT scan were available.

Nevertheless, it is still possible that selection bias may have occurred, as patients without clinical or radiological evidence of RR are less likely to be referred to a tertiary centre. Consequently, our dataset is still likely to overrepresent RR compared to the general population.

In order to further refine our RR rates, we conducted a comparative analysis between two groups of patients. The first group (group 1) consisted of individuals with sings of potential RR on PAN, while the second group (group 2) comprised those without such suspicious facts. Our findings revealed that RR was present in 24.3% of patients in group 1, in comparison to 16.3% in group 2. Notably, the latter group exhibited a lower prevalence of RR compared to the other studies.

To account for the aforementioned dependency of observations when examining multiple M3 per patient, we performed a patient level-analysis. For every patient, we only considered one of the M3. RR was still measurably more common for M3 showing superimposition in the PAN compared to those without (31.4% for group 1 and 17.9% for group 2) Also, the level of RR according to Ericson et al. was higher for M3 showing superimposition in the PAN.

Thus, both the per molar-analysis as well as the per patient-analysis confirm that RR is very common even without restrictive selection criteria.

Our data indicate that conventional (PAN) imaging is an imprecise method for radiological assessment, with a high frequency of false positive and false negative diagnoses. Furthermore, our results suggest that the overlap of a retained M3 and its adjacent M2 on PAN does not allow for the prediction of RR. Likewise the absence of overlap does not rule out RR. However, the degree of RR appears to be higher when there is a visible overlap of the molars. It is regrettable that even moderate to severe RR (potentially associated with a limited prognosis of the M2) cannot be safely excluded with conventional PAN. Consequently, if advanced RR is not detected prior to the M3 removal, additional loss of M2 may occur as collateral damage.

In a study by Oenning et al., it was found that periapical radiography (PAN) could only image less than 25% of the RR seen on CBCT [27]. Our study corroborates these findings, as we also detected RR in less than 5% of cases on PAN but found a rate of approximately 20% on CBCT. Specifically, out of 40 moderate to severe RR cases, only 3 were diagnosed in the PAN. In conclusion, the data presented here and those of Oenning and others support the use of CBCT imaging prior to M3 removal. However, in order to balance the positive impact of diagnostic improvement against the negative effects of radiation exposure, additional parameters should be considered to avoid unnecessary radiological screening and to target patients at risk of RR and specifically those with a clinically relevant degree of RR.

The grading of RR according to the Ericson classification [2] allows the assessment of clinical relevance, as mild RR usually does not affect M2 prognosis. Therefore, we conducted a search for predictive parameters not only for the prevalence of RR but also for the severity of RR.

Of the systemic parameters considered, only age appears to be a predictive variable for RR. This seems reasonable, since mild and stagnant RR can remain undetected for decades and RR is an irreversible phenomenon that can be either progressive or stagnant, but never regressive. Accordingly, patients with moderate or severe RR were more than ten years older than those with no or mild RR. Orthodontic treatment or gender did not appear to influence RR, either in terms of prevalence or severity.

The rate of RR in patients under 28 years of age was comparable to that found in group 2 (17.4% vs. 16.3%), whereas the rate of RR in patients over 28 years of age was 27.7%, which is even higher than the average rate of RR found in group 1 (24.3%). It may therefore be appropriate to consider routine CBCT imaging in patients over the age of 28 in order to rule out M2-RR prior to M3 removal. This is because a patient age of 28 years and older seems to increase the RR rate at least as much as superimposition in PAN. In the per-patient analysis, the probability of RR was found to be significantly higher for patients older than 28 years compared to those younger than 28 years (*p* = 0.022). However, the rate of RR for all patients younger than 28 years was slightly higher than the rate of total RR in group 2 (20.5% vs. 17.9%), while the rate of RR for patients older than 28 years was very close to the rate of RR found in group 1 (31.1% vs. 31.4%). This is congruent with the findings from the per molar analysis, which further reinforces the recommendation for routine CBCT examination of patients aged 28 years or older.

In accordance with the findings of the preceding studies, our analysis revealed that the angle of inclination, the depth of retention of M3 and the contact point of M2 and M3 were ascertained as predictive local parameters for RR. Our data indicate that orthoaxially retained M3 without overlap exhibited the lowest risk of RR (13.9%), followed by mesioangulated M3 without superimposition (18.9%) and orthoaxially retained M3 with overlap (19.7%). The highest rate of RR was found in mesioangulated M3 with superimposition of M2 (27.3%). The superimposition appears to have an impactful effect on the severity of the RR, with a maximum of 3% of moderate/severe RR observed when there is no superimposition in the PAN, compared to a maximum of 7.2% when there is superimposition.

It seems reasonable to assume that the angle of the retained M3 determines the eruption direction, with mesioangulation forcing the M3 to erupt towards the adjacent M2. Conversely, orthoaxially retained M3s pass the M2 with little or no contact. Similarly, a greater depth of retention and a more apical point of contact would most likely increase the risk of RR.

The angulation and depth of retention of the M3 may also be influenced by the vertical facial dimension. In 2020, Lo Guidice et al. evaluated cortical bone thickness in patients with different vertical facial dimensions using CBCT imaging. In particular, they examined condylar cortical bone thickness for different types of vertical facial dimension. They found higher condylar bone thickness in patients with a more vertical facial dimension [36]. In contrast, patients with hypodivergent facial growth patterns tend to have an increased cortical bone thickness in certain areas of the mandibular body. It should be noted that we did not include facial growth patterns in our analysis. However, future studies might address the potential influence of cortical bone thickness on RR occurrence and severity.

Taken together, our results confirm that RR is not a rare phenomenon and that PAN-imaging alone is not sufficient to depict RR, as 16.3% of M2 showed RR on CBCT despite the lack of superimposition on PAN. 2% of M2 even showed moderate to severe RR and are therefore likely to have a limited long-term prognosis. Expanding the indication for CBCT imaging prior to M3 surgery may be useful to avoid M3 removal without knowing about M2 damage due to RR. Specifically, CBCT-imaging may be helpful to rule out RR in severely retained, mesioangulated M3 with a rather deep contact zone to M2 in patients aged 28 years and older.

CBCT imaging may be used to generate 3D models and calculate volumetric data of M2 roots [37], which may help to estimate clinical relevance even of mild RR, as the current Ericsson classification only considers the maximum depth of RR and not the total loss in root volume.

We are aware that our study has some weaknesses.

The present study was conducted in a tertiary oral and maxillofacial surgery centre, which typically treats patients with more severe cases. In order to mitigate this effect, a separate analysis was conducted of M3 with (Group 1) and without (Group 2) potential signs of resorption in the PAN. It seems reasonable to posit that this approach allows us to make a more accurate estimation of the prevalence of root resorption in the general population, rather than focusing exclusively on high-risk patients, as is often the case in other studies. Nevertheless, it is possible that the observed prevalence and severity of root resorptions may be overestimated due to a potential selection bias. But even if only the cases of medium and high-grade resorption are taken into account, it can be assumed that in Germany alone there are around 20,000 patients per year in whom resorption can have an impact on the decision to remove wisdom teeth.

The use of M3 as the unit of research may also result in an overestimation of potential risk factors due to the accumulation of dependent observations. Conversely, a solely patient-based evaluation may underestimate the relevance of risk factors due to the exclusion of a subset of the available data. Although widely accepted in exploratory analyses, abstaining form alpha-adjustment may result in a higher false positive rate of hypotheses. Nevertheless, adjustment strategies like the Bonferroni method reduce the probability of rejecting null hypotheses that are in fact incorrect [38].

Due to the retrospective nature of our study, we cannot comment on the progression of RR and especially the long-term outcome of partially resorbed M2. Longitudinal follow-up would be required to monitor the evolution of RR in patients who decided to keep their M3 despite mild M2 RR.

We are also aware, that the threshold age of 28 years for CBCT diagnosis is a rough approximation based on a patient cohort in which almost two thirds of the patients were 25 years or younger. Therefore, a larger sample size including more patients at older ages may be helpful in a further confirmatory study.

## Data Availability

The data that support the findings of this study are available from the corresponding author upon reasonable request.

## Declarations

**Human ethics and consent to participate declaration**.

Not applicable, as approved by the institutional review board of Ruhr University, Bochum, Germany (Reg.-Nr. 18-6652-BR) in view of the retrospective nature of the study and all the procedures being performed were part of the routine care.

### Dual publication

The results/data/figures in this manuscript have not been published elsewhere, nor are they under consideration (from you or one of your Contributing Authors) by another publisher.
